# Non-stress test as a predictor for high-risk pregnancy in the third trimester using fetal color Doppler

**DOI:** 10.6026/973206300220152

**Published:** 2026-01-31

**Authors:** Neelam Tejwani, Neha Chaitanya, Razia Sultana, Sachin Parmar

**Affiliations:** 1 Department of Obstetrics and Gynaecology RD Gardi Medical College CRGH Hospital Ujjain, Madhya Pradesh, India; 2 Department of Obstetrics and Gynaecology Lokmanya Tilak Municipal General Hospital and Lokmanya Tilak Municipal Medical College, Sion, Mumbai, India; 3 Department of Community Medicine, V.K.S. Government Medical College, Neemuch, Madhya Pradesh, India

**Keywords:** Non-stress test, Doppler ultrasound, high-risk pregnancy, fetal distress, perinatal outcome

## Abstract

Maternal and fetal complications in high risk pregnancies are among the leading causes of perinatal morbidity and mortality worldwide. Therefore, it is of interest to evaluate the utility of the non-stress test (NST) and Doppler ultrasound of the umbilical artery in expectancies of unfavourable perinatal outcomes in high-risk pregnancies. Data shows that abnormal NST and Doppler results were associated with high fetal distress, cesarean sections, small birth weight and NICU admission with Group D, (abnormal NST and Doppler), representing the poor results. The research indicates the interest in reducing the burden of high-risk pregnancies, where it was found that the combination of these tools works well as an early identification and intervention procedure.

## Background:

Maternal or fetal complications during high risk pregnancy are significant contributors to perinatal morbidity and mortality all over the world. Early detection and management of these pregnancies are important toward the evasion of poor neonatal outcomes. Non-stress test (NST) and Doppler ultrasound are commonly used antenatal surveillance modalities to assess fetal well-being in the third trimester and especially in pregnancy where women experience pre-eclampsia, intrauterine growth restriction (IUGR) and gestational diabetes mellitus (GDM) [1]. NST is a non-invasive test that evaluates heart rate (FHR) accelerations during fetal movements, to gain some understanding of functional status of the fetus autonomic nervous system. A non-reactive NST would be a matter of concern and may amount to the presence of the fetal hypoxia requiring other tests to be done to confirm the presence or absence thereof [2]. Nevertheless, NST, when used alone, has limits to foreseeing poor perinatal outcomes, which prompted the use of an adjunct Doppler ultrasound test to be added to it [3]. Umbilical arteries Doppler ultrasound analyses the blood flow impedance and placental vascular resistance of the fetus and is a good fingerboard of the oxygen hoard of the fetus. An increase in resistance to the umbilical artery is related to placental insufficiency and fetal hypoxia that is quite common in IUGR pregnancies [3]. Deviant Doppler results and more specifically high systolic/diastolic (S/D) ratios, absent/reversed end-dialtolic flow and higher pulsatile indices are associated with inappropriate perinatal outcome, such as fetal stress, hypoxemia of newborn admitted to newborn intensive care unit (NICU) and stillbirths [4]. The combination of the NST and Doppler ultrasound has been the topic of a few studies in which its use has been made to increase the predictability of fetal surveillance. A review by Chauhan *et al.* established that NST combined with umbilical artery Doppler enhanced detection of fetal distress and indicate of the requirement of emergency obstetric procedures. In a similar study, the meta-analysis by Alfirevic *et al.* found out that Doppler ultrasound is better than conventional fetal monitoring in reducing perinatal deaths particularly among high-risk pregnancies [5]. Since the issue of fetal distress and poor perinatal outcomes is highly critical, it is of interest to evaluate the predictive capacities of NST alongside umbilical artery Doppler in high-risk pregnancies. Therefore, it is of interest to assess and delineate the predictive capacity of the non-stress test in conjunction with umbilical artery Doppler ultrasound for the identification of adverse perinatal outcomes in high-risk pregnancies during the third trimester.

## Materials and Methods:

## Study setting and population:

The research work was carried out in the Department of Obstetrics and Gynaecology in Ruxmaniben Deepchand Gardi Medical College, Ujjain, which was a unit of charitable trust hospital and research centre. The institute liaises with all over the world research institutes and has an 800 bed hospital and on average does 2000 obstetric and gynecological cases in house per month. Antenatal women were the population of the study, those in outpatient clinics or admission associated with conditions included pre-eclampsia and intrauterine growth restriction (IUGR). The hospital is mainly rural and socioeconomically deprived. This prospective observational study has been carried out between November 2020 and October 2021.

## Participant recruitment:

The number of women recruited as antenatal was 182, arriving at these numbers using particular inclusion and exclusion factors. The inclusion criteria included hypertension in pregnancy, diabetes superimposed on pregnancy, IUGR, post-term pregnancy, deviations in ingestion of liquor, adverse obstetric history, a reduction in fetal movements, severe anemia, Rh isoimmunization and increased maternal age (>=35 years). Gestational age <30 weeks, anterior hemorrhage, eclampsia, uncomplicated multiple gestations, ruptured membranes, congenital anomalies, malpresentations, prior LSCS, cephalopelvic disproportion or sedation of the mother 24 hours prior to the test were used as the exclusion criteria.

## Collection and analysis of data:

Detailed demographic and obstetric, medical and family history was secured. Clinical tests involved general tests, body system tests and obstetric tests like measurement of the fundal height, heart rate monitoring of the baby and the growth of the baby. Regular antenatal tests were done, which included hemoglobin estimation, blood grouping, random blood sugar and infections screening. To establish the pregnancy at the right gestation period, biometric measurements, presence of amniotic fluid index, fetal position and congenital malformations, obstetric ultrasonography was done.

## Evaluation Doppler and NST:

The color Doppler evaluated fetal circulation by paying attention to the umbilical and middle cerebral arteries. Ductusvenosus flow reversal was an indicator of severe fetal compromise as was the recorded pulsatility index (PI), resistance index (RI) and systolic- diastolic ratio (S/D). NSTs were carried out through at least 20 minutes and interpreted on ACOG rules. According to Doppler and NST results, study subjects were placed in four categories of: Group A (normal NST and Doppler), Group B (normal NST and abnormal Doppler), Group C (abnormal NST and normal Doppler) and Group D (abnormal NST and Doppler).

## Outcome measures:

The adverse perinatal outcomes were low birthweight (below the 10th percentile), birth by cesarean section due to fetal distress, perinatal death, low Apgar score at 5 minutes (below 7) and admission to the NICU because of complications like birth asphyxia, sepsis, respiratory distress syndrome, or hypoglycemia. There was an evaluation of the maternal outcome according to the mode of delivery and the gestational age at delivery.

## Data analysis:

The data were statistically analyzed and SPSS software was carried out. Sensitivity, specificity, the positive and negative predictive value of results and rates of the false-positive and false-negative results as well as overall accuracy were computed. P-values were used to establish statistical significance and results put across as tables and graphs.

## Ethical approval:

The research undertaken was based on ethical procedures having met the clearances of the Institutional Ethics Committee (IEC Ref. No-48/2021). There was confidentiality and there were no foreseen identifiable risks to the participants.

## Results:

The distribution of cases reveals that the majority of individuals were aged between 26 and 30 years 77 (42.3%), followed by 21-25 years 56 (30.8%), while those aged ≤20 and >35 years constituted the smallest proportion 5 (2.7%) each. Among risk factors, anemia was the most prevalent 42 (23.1%), followed by pregnancy-induced hypertension 26 (14.3%), intrauterine growth restriction 21 (11.5%), oligohydramnios 21 (11.5%) and post-dated pregnancies 22 (12.1%). Diabetes mellitus 11 (6.0%), hyperthyroidism 18 (9.9%) and Rh-negative status 9 (4.9%) were also observed. Doppler findings indicated abnormal readings in 66 (36.3%) of cases, while 116 (63.7%) had normal results. Similarly, non-reactive NST was found in 72 (39.6%), with the remaining 110 (60.4%) classified as reactive. Regarding delivery mode, normal delivery was more frequent 115 (63.2%), whereas LSCS accounted for 67 (36.8%). Group A had the highest proportion 98 (53.8%), followed by Group D 54 (29.7%), while Group B comprised the smallest fraction 12 (6.6%).The distribution of perinatal and neonatal parameters among study groups is presented in Table 1, while Table 2 illustrates the correlation of Doppler and NST findings with perinatal outcomes.

## Discussion:

This study finding suggests that the NST and Doppler ultrasound of the umbilical artery combined application is a good predictor of adverse perinatal outcome in risky pregnancies. Abnormal NST and Doppler had significant association with higher frequencies of cesarean delivery, fetal distress, low score of APGAR scores and admissions into the NICU. The results can be aligned with past studies that promoted the significance of fetal surveillance in pregnancies at risk to avoid birth complications of the newborn. NST and Doppler ultrasound used as fetal monitoring methods are important in the determination of placental insufficiency and fetal distress. The NST mainly estimates the fetal well-being via fetal heart rate variability responding to fetal movements whereas the Doppler studies offer us with information on fetal circulation and evaluate resistance indices of the umbilical and middle cerebral arteries. Doppler abnormalities, such as an abnormal systolic-diastolic (S/D) ratio, high resistance index (RI) and reversed end-diastolic blood flow, are attributed to fetal hypoxia and poor neonatal outcome [6]. Various studies have found Doppler abnormalities to be predictive of perinatal mortality and morbidity in high-risk pregnancies, especially those of intrauterine growth restriction (IUGR) and pre-eclampsia [7, 8]. The findings of this study revealed that Group D (abnormal NST and Doppler) recorded the highest rate of poor perinatal outcomes with respect to fetal distress (33.3%), a low Apgar score (<7 at 5 minutes in 72.2%) and death causing neonatal deaths (5.6%). This is consistent with the findings reported by earlier studies meaning that a combination of fetal testing (NST and Doppler) is a superior predictor of fetal compromise as compared to either test alone [9]. A study conducted by Grivell *et al.* indicated that abnormal Doppler resulted in elevated rates of cesarean delivery because of non-reassuring fetal conditions [9]. Our results showed that cases of abnormal Doppler and NST results had a significant increase in the rates of cesarean section (87% in Group D). Abnormal fetal assessments also played an important role in neonatal outcomes such as NICU admissions this finding aline with the study done by Ayyuub *et al.* [10] The majority of neonates with variations in NST and Doppler values (66.7%) were admitted to NICU and this percentage corresponds to the results obtained by Alfirevic *et al.* who discovered that an abnormality in Doppler measurements is associated with a significant relationship with increased NICU admission and neonatal mortality in the instance of growth restriction [1]. Additionally, the occurrence of meconium-stained liquor was higher among the groups of neonates with non-reactive NST, as it indicates fetal distress and hypoxemia that are associated with increased [11]. In spite of the solid results, there are limitations to this study. It only sampled one tertiary care facility and this might be a limitation as far as its generalizability is concerned. Moreover, other fetal monitoring equipment e.g. biophysical profile scoring and contraction stress test were not included in the study design. The role of combined usage of other modalities of fetal monitoring in a high-risk pregnancy still needs to be studied in the future and implement further research to refine the use of monitoring in high-risk pregnancy.

## Conclusion:

The combination of the non-stress test and umbilical artery Doppler ultrasound is a useful method for forecasting unfavorable perinatal outcomes in high-risk pregnancies. Fetal distress, low birth weight, cesarean delivery and NICU admissions are strongly correlated with abnormal results on both tests. By combining these approaches, fetal compromise can be detected earlier, allowing for prompt interventions and better perinatal outcomes.

## Figures and Tables

**Table 1:** Distribution of perinatal and neonatal parameters among study groups

**Parameter**	**Group A (N, %)**	**Group B (N, %)**	**Group C (N, %)**	**Group D (N, %)**	**p-value**
Liquor					0.413
Clear	66 (67.3%)	8 (66.7%)	10 (55.6%)	41 (75.9%)	
Meconium-stained	32 (32.7%)	4 (33.3%)	8 (44.4%)	13 (24.1%)	
Mode of Delivery					0
Normal	83 (84.7%)	7 (58.3%)	18 (100.0%)	7 (13.0%)	
LSCS	15 (15.3%)	5 (41.7%)	0 (0.0%)	47 (87.0%)	
APGAR at 1 min					0
< 7	7 (7.1%)	11 (91.7%)	1 (5.6%)	43 (79.6%)	
>= 7	91 (92.9%)	1 (8.3%)	17 (94.4%)	11 (20.4%)	
APGAR at 5 min					0
< 7	7 (7.1%)	12 (100.0%)	1 (5.6%)	39 (72.2%)	
>= 7	91 (92.9%)	0 (0.0%)	17 (94.4%)	15 (27.8%)	
NICU Admission					0
Yes	7 (7.1%)	9 (75.0%)	1 (5.6%)	36 (66.7%)	
No	91 (92.9%)	3 (25.0%)	17 (94.4%)	18 (33.3%)	
Fetal Distress					0
Yes	4 (4.1%)	6 (50.0%)	4 (22.2%)	18 (33.3%)	
No	94 (95.9%)	6 (50.0%)	14 (77.8%)	36 (66.7%)	
Neonatal Death					0.057
Yes	0 (0.0%)	1 (8.3%)	0 (0.0%)	3 (5.6%)	
No	98 (100.0%)	11 (91.7%)	18 (100.0%)	51 (94.4%)	

**Table 2:** Correlation of Doppler and NST findings with perinatal outcomes

**Parameter**	**Category**	**Clear (N, %)**	**Meconium-stained (N, %)**	**Total (N, %)**	**Chi-Square**	**p-value**
Doppler vs Liquor	Abnormal	49 (39.2%)	17 (29.8%)	66 (36.3%)	1.48	0.222
	Normal	76 (60.8%)	40 (70.2%)	116 (63.7%)		
NST vs Liquor	Non-Reactive	51 (40.8%)	21 (36.8%)	72 (39.6%)	0.256	0.613
	Reactive	74 (59.2%)	36 (63.2%)	110 (60.4%)		
Doppler vs Mode of Delivery	Abnormal	52 (77.6%)	14 (12.2%)	66 (36.3%)	78.43	0
	Normal	15 (22.4%)	101 (87.8%)	116 (63.7%)		
NST vs Mode of Delivery	Non-Reactive	47 (70.1%)	25 (21.7%)	72 (39.6%)	41.49	0
	Reactive	20 (29.9%)	90 (78.3%)	110 (60.4%)		
Doppler vs APGAR at 1 min	Abnormal	54 (87.1%)	12 (10.0%)	66 (36.3%)	105.12	0
	Normal	8 (12.9%)	108 (90.0%)	116 (63.7%)		
NST vs APGAR at 1 min	Non-Reactive	44 (71.0%)	28 (23.3%)	72 (39.6%)	38.79	0
	Reactive	18 (29.0%)	92 (76.7%)	110 (60.4%)		
Doppler vs APGAR at 5 min	Abnormal	51 (86.4%)	15 (12.2%)	66 (36.3%)	95.09	0
	Normal	8 (13.6%)	108 (87.8%)	116 (63.7%)		
NST vs APGAR at 5 min	Non-Reactive	40 (67.8%)	32 (26.0%)	72 (39.6%)	29.11	0
	Reactive	19 (32.2%)	91 (74.0%)	110 (60.4%)		
Doppler vs NICU Admission	Abnormal	45 (84.9%)	21 (16.3%)	66 (36.3%)	76.54	0
	Normal	8 (15.1%)	108 (83.7%)	116 (63.7%)		
NST vs NICU Admission	Non-Reactive	37 (69.8%)	35 (27.1%)	72 (39.6%)	29.61	0
	Reactive	16 (30.2%)	94 (72.9%)	110 (60.4%)		
Doppler vs Fetal Distress	Abnormal	24 (75.0%)	42 (28.0%)	66 (36.3%)	25.2	0
	Normal	8 (25.0%)	108 (72.0%)	116 (63.7%)		
NST vs Fetal Distress	Non-Reactive	22 (68.8%)	50 (33.3%)	72 (39.6%)	13.83	0
	Reactive	10 (31.3%)	100 (66.7%)	110 (60.4%)		
Doppler vs Neonatal Death	Abnormal	4 (100.0%)	62 (34.8%)	66 (36.3%)	7.188	0.007
	Normal	0 (0.0%)	116 (65.2%)	116 (63.7%)		
NST vs Neonatal Death	Non-Reactive	3 (75.0%)	69 (38.8%)	72 (39.6%)	2.148	0.143
	Reactive	1 (25.0%)	109 (61.2%)	110 (60.4%)		
Doppler vs NST Findings	Non-Reactive	54 (81.8%)	18 (15.5%)	72 (39.6%)	77.33	0
	Reactive	12 (18.2%)	98 (84.5%)	110 (60.4%)		
